# Materials based on biodegradable polymers chitosan/gelatin: a review of potential applications

**DOI:** 10.3389/fbioe.2024.1397668

**Published:** 2024-08-02

**Authors:** Aref Yarahmadi, Behrooz Dousti, Mahdi Karami-Khorramabadi, Hamed Afkhami

**Affiliations:** ^1^ Department of Biology, Khorramabad Branch, Islamic Azad University, Khorramabad, Iran; ^2^ Department of Mechanical Engineering, Khorramabad Branch, Islamic Azad University, Khorramabad, Iran; ^3^ Cellular and Molecular Research Centre, Qom University of Medical Sciences, Qom, Iran; ^4^ Nervous System Stem Cells Research Center, Semnan University of Medical Sciences, Semnan, Iran; ^5^ Department of Medical Microbiology, Faculty of Medicine, Shahed University, Tehran, Alborz, Iran

**Keywords:** chitosan, gelatin, antimicrobial, antioxidant, biodegradable polymers

## Abstract

Increased mass manufacturing and the pervasive use of plastics in many facets of daily life have had detrimental effects on the environment. As a result, these worries heighten the possibility of climate change due to the carbon dioxide emissions from burning conventional, non-biodegradable polymers. Accordingly, biodegradable gelatin and chitosan polymers are being created as a sustainable substitute for non-biodegradable polymeric materials in various applications. Chitosan is the only naturally occurring cationic alkaline polysaccharide, a well-known edible polymer derived from chitin. The biological activities of chitosan, such as its antioxidant, anticancer, and antimicrobial qualities, have recently piqued the interest of researchers. Similarly, gelatin is a naturally occurring polymer derived from the hydrolytic breakdown of collagen protein and offers various medicinal advantages owing to its unique amino acid composition. In this review, we present an overview of recent studies focusing on applying chitosan and gelatin polymers in various fields. These include using gelatin and chitosan as food packaging, antioxidants and antimicrobial properties, properties encapsulating biologically active substances, tissue engineering, microencapsulation technology, water treatment, and drug delivery. This review emphasizes the significance of investigating sustainable options for non-biodegradable plastics. It showcases the diverse uses of gelatin and chitosan polymers in tackling environmental issues and driving progress across different industries.

## 1 Introduction

The management of plastic waste presents a significant environmental challenge in contemporary society. The widespread utilization of plastics in various aspects of daily life, coupled with the escalation of mass production, has led to significant environmental consequences (Moharir and Kumar, 2019; PanSu et al., 2020). As a result, these concerns contribute to the increasing risk of climate change caused by releasing carbon dioxide from the incineration of non-biodegradable traditional polymers like polyethylene, polyvinylchloride, and polypropylene. It is also essential to consider the environmental impacts associated with the production processes of biodegradable alternatives (Amulya et al., 2021). Biodegradable polymers are developing as a sustainable substitute for non-biodegradable polymer materials across various applications (Ahmed et al., 2023). The most effective approach for addressing non-biodegradable plastic waste involves substituting economically inefficient materials with biodegradable polymers for recycling or reutilization, given their environmentally sustainable properties (Flury and Narayan, 2021). A biodegradable polymer is a substance that can be broken down by microorganisms, as well as environmental factors like temperature and oxygen, into less complex components that do not pose harm to the ecosystem (Yin and Yang, 2020; Wu et al., 2021). Moreover, biodegradable polymers are utilized in various industries based on their price, ability to absorb moisture, accessibility, mechanical properties, antibacterial characteristics, thermal resistance, and compatibility with living organisms (Christian, 2020; Vieira et al., 2022). Chitosan, a polysaccharide, and gelatin, a protein, are two biodegradable polymers that have demonstrated diverse utility in various fields such as packaging, agriculture, wastewater treatment, drug delivery, orthopedics, wound dressings, tissue engineering, and other applications (Wang et al., 2021; Ebhodaghe, 2022; Sethi and Kaith, 2022). Chitosan, specifically, possesses antimicrobial characteristics that can prolong the storage duration of food items by suppressing the proliferation of bacteria and fungi. In contrast, gelatin is an effective barrier material that protects against oxygen, moisture, and various contaminants, rendering it a viable choice for food packaging applications (Flórez et al., 2022; Lu et al., 2022). Also, they have demonstrated potential in a range of medical applications within the healthcare sector, including but not limited to wound dressings, drug delivery mechanisms, and tissue engineering. For instance, chitosan has been employed in wound dressings because of its hemostatic and antimicrobial characteristics. In contrast, gelatin has been applied in tissue engineering as a support structure for promoting cell proliferation (Bello et al., 2020; Moeini et al., 2020; Ding et al., 2021; Lukin et al., 2022). Numerous studies have shown that combining chitosan and gelatin produces a high-quality and uniform film (Xu D. et al., 2021a; Roy and Rhim, 2021). Chitosan and gelatin possess additional advantageous characteristics, including the ability to minimize harm to non-targeted cells or tissues and inhibit the enzymatic breakdown of medications (Liu Y. et al., 2022a; Battogtokh et al., 2022; Zhu et al., 2022). Because of these qualities, gelatin and chitosan are excellent materials for biological imaging and diagnostics, medication delivery systems, and cancer therapy (Wegrzynowska-Drzymalska et al., 2022; Zhou et al., 2022). Moreover, previous studies have indicated that the gradual decomposition of gelatin and chitosan nanoparticles (NPs) contributes to a regulated and sustained release of drugs. This is attributed to the solid positive surface charges of these NPs, which serve as stable vehicles for delivering substances to specific locations within the human body (Nagpal et al., 2010; Sahoo et al., 2015; Garg et al., 2019). Nevertheless, chitosan and gelatin possess constraints related to their physicochemical stability. Scholars persist in investigating strategies to address these obstacles by implementing modifications and incorporating other materials to enhance their efficacy (Rodríguez-Rodríguez et al., 2020; Ebhodaghe, 2022).

The objective of this review is to provide an overview of the biopolymers chitosan and gelatin, as well as to outline the latest advancements in their utilization as animal-derived products in food, pharmaceuticals, and medicine.

### 1.1 Chitosan

**FIGURE 1:**
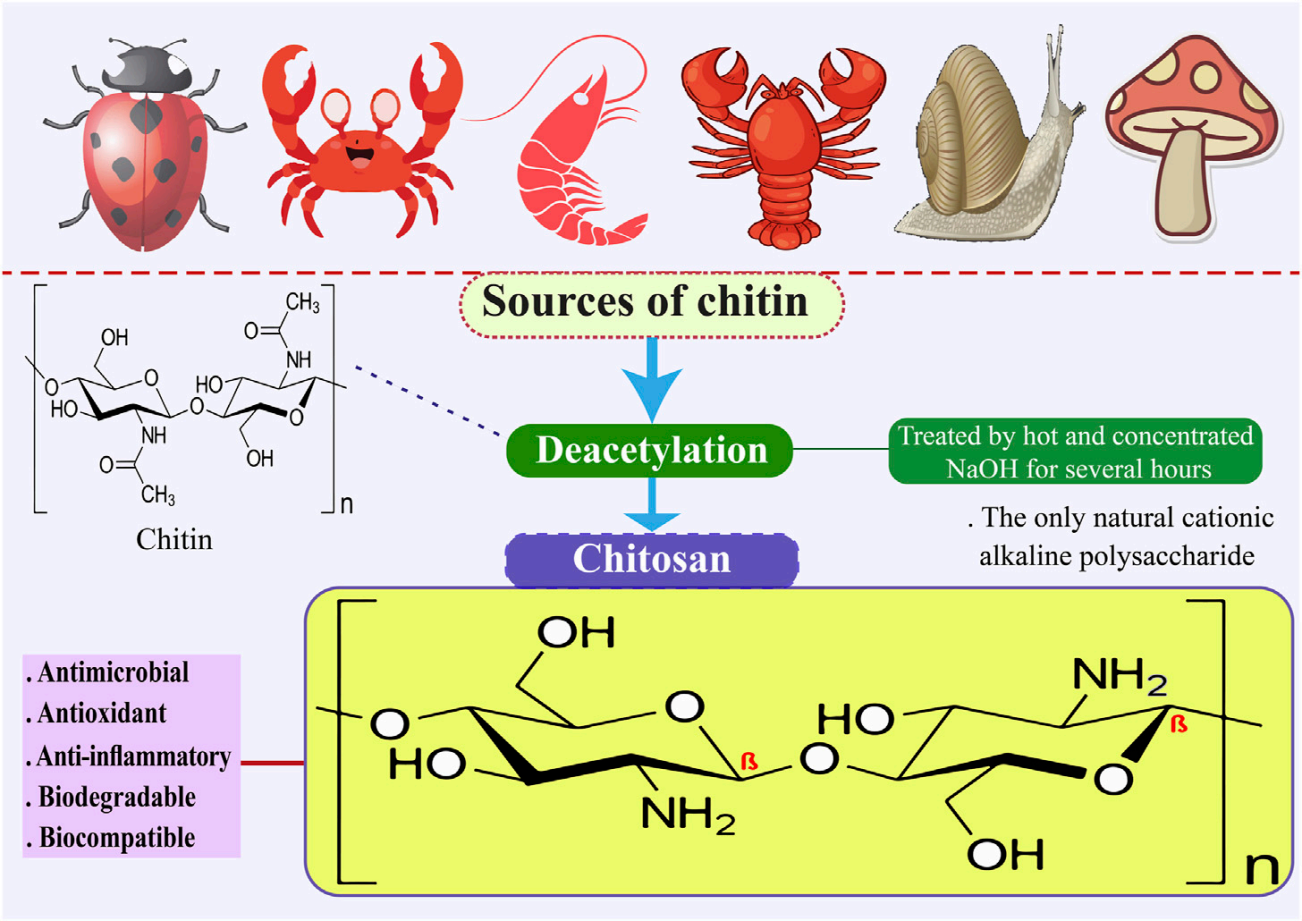
Sources of chitin and structure of chitosan.

Chitosan is the only naturally occurring cationic alkaline polysaccharide. Its scientific composition is 2-amino-2-deoxy-D-glucose. Chitosan can be synthesized through the deacetylation of chitin using NaOH, as illustrated in Figure 1, and through fermentation processes involving certain microbial cultures. Shrimp, crab, and bug shells are the primary sources of chitin (Mohan et al., 2020; Mulyani et al., 2020; Tan et al., 2020). Chitosan is a readily accessible and cost-effective polysaccharide with semi-crystalline properties, primarily soluble in mild organic acids, including lactic, acetic, citric, tartaric, formic, and malic acids (Cazón et al., 2021; Bhowmik et al., 2022). Chitosan, derived from various sustainable sources, is a bio-resource known for its exceptional antibacterial and antioxidant properties, as well as its ability to inhibit enzymes. It is considered safe for consumption, environmentally friendly, and biodegradable. Consequently, extensive research is being conducted on chitosan across various sectors, such as food science, pharmaceuticals, environmental conservation, chemical engineering, cosmetics, agriculture, and textiles (Wang et al., 2020; Kou et al., 2021). Chitosan has been the subject of extensive research across various applications and sectors owing to its antibacterial, antioxidant, biodegradable, enzyme-inhibitory, and biocompatible properties (Jiménez-Gómez and Cecilia, 2020; Ahghari et al., 2022; Bashir et al., 2022; Liu T. et al., 2022b). Chitosan is a biopolymer that shows promise for food packaging applications due to its capacity to suppress the growth of Gram-negative and Gram-positive bacteria, yeasts, and food-borne filamentous fungi. Furthermore, it functions as an antimicrobial substance, a vehicle for delivering antimicrobial agents and prebiotics that can improve the body’s ability to resist colonization by harmful pathogens (Jiang et al., 2023). The fascination with the structure and utilization of chitosan can be traced back to the 19th century. In 1859, Rouget was the first to explore the deacetylated variations of chitin, the parent natural polymer found abundantly in nature. Presently, chitosan has obtained Generally Recognized as Safe status from the U.S. Food and Drug Administration (Oleksy et al., 2023). Chitosan is recognized for its diverse advantageous characteristics, such as its capacity to adhere to fats and cholesterol within the gastrointestinal (GI) system, potentially leading to a decrease in cholesterol levels and facilitating weight management (Huang et al., 2020). Nevertheless, its drawbacks encompass a diminished capacity for dissolving in water, resulting in the formation of a firm crystal structure. Furthermore, its elevated water vapor permeability proves unsuitable for environments with high humidity levels (Elsabee, 2013; Candir et al., 2018; Chaudhary et al., 2020). Table 1 shows that chitosan can be used in many industries.

**TABLE 1:** Various applications of chitosan.

Applications	Examples	References
Tissue engineering	Repair of scaffolds, regeneration of bones and tissues, regeneration of sulphate sponges in bone, diabetes treatment, development of artificial pancreas, cartilage regeneration, skin tissue regeneration, cardiac tissue regeneration	Islam et al. (2020), Kołodziejska et al. (2021), Kim et al. (2023)
Pharmaceutical and biomedical materials	Drug delivery systems, treating burns, surgical structures, dental repair and treatment, lenses for eyes, artificial skin, dialysis of blood, accelerated wound healing, antitumor and antibiotic uses, and synthetic blood vessels	Iacob et al. (2021), Khalaf et al. (2023), Almajidi et al. (2024)
Cosmetics	Skin and hair care products	Guzmán et al. (2022), Kulka and Sionkowska (2023)
Food and feed additives	Extension of natural flavor, color stabilization in foods, lipid absorption reduction, food and beverage de-acidification, antioxidant and food preservation, stabilizing agent, thickening agent, controlling agent, additives in livestock and fish food, manufacture of dietary fibers	Muzzarelli and De Vincenzi (2020), Anggraeni et al. (2022)
Water engineering	Treatment of waste water, dye removal from water, removal of pesticides and ions from water, removal of heavy metals from water, removal of petroleum products from water, color removal from textile waste waters, removal of dyes from effluents	Ahmed et al. (2020), Bhatt et al. (2023)
Food packaging	Covering various fruits and vegetables, spraying chitosan on food, preparing a film, controlling food contaminating microbes, covering meat, fish, chicken, increasing the shelf life of food	Kumar et al. (2020a), Priyadarshi and Rhim (2020), Flórez et al. (2022)
Gene therapy	It delivers numerous genes employed in gene therapy, siRNA, and cancer therapy technologies	Wu et al. (2020), Reshad et al. (2021)
Agriculture	Seed coating, excellent film coating with antimicrobial activities, removal of pesticides and herbicides from soil and water, preservation of post harvested foods, enhancing soil quality, enhancing plant growth	Faqir et al. (2021), Zhang et al. (2022a), Hidangmayum and Dwivedi (2022)

Depending on the degree of deacetylation and the chitin source, the molar mass of commercially available chitosan products varies greatly, usually ranging from 50 kDa to over 1,000 kDa. Chitosan exhibits solubility in acidic to neutral solutions due to its pKa value of around 6.5 (Kumar et al., 2004; Ogawa et al., 2004; de Alvarenga, 2011). Since chitosan is mainly derived from the shells of crustaceans, nations with sizable seafood industries dominate chitosan manufacturing. China, India, and Japan are the top manufacturers; these countries have set up extensive production plants to fulfill the demand from throughout the world (de Alvarenga, 2011; Yadav et al., 2019; Santos et al., 2020; Huq et al., 2022). Hydrogen bonding between the polymer chains of chitosan plays a crucial role in its structural stability and contributes to its distinct physical features (Ogawa et al., 2004; Chen et al., 2018). Furthermore, lysozyme—an enzyme in human tears, saliva, and other physiological fluids—acts as the primary biodegradation agent for chitosan. Chitosan is an advantageous material for biomedical applications because of its enzymatic breakdown, which produces non-toxic byproducts (Lončarević et al., 2017; Desai et al., 2023).

### 1.2 Gelatin

**FIGURE 2:**
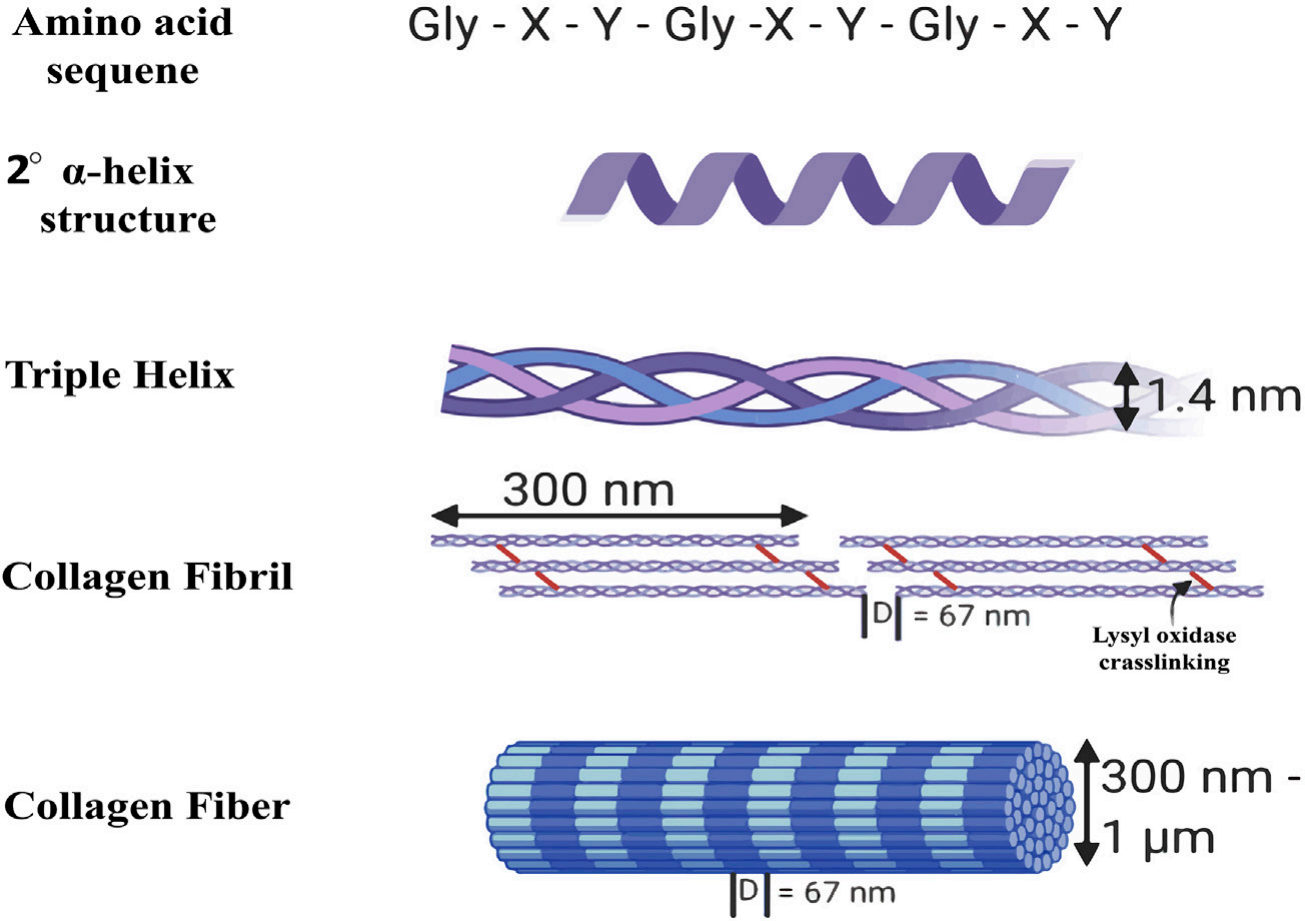
Structure of collagen (Walimbe and Panitch, 2020).

Gelatin is a naturally occurring polymer derived from the hydrolytic breakdown of collagen protein. Its unique amino acid composition imparts various medicinal advantages Figure 2 (Kumosa et al., 2018). The substance is a transparent gel that exhibits fissures upon desiccation due to the degradation of collagen within tissues and skeletal structures (Alipal et al., 2021). Following collagen isolation, gelatin can be obtained through two methods: acid hydrolysis or alkaline hydrolysis (Bello et al., 2020; C Echave et al., 2017; Tabata and Ikada, 1998). Collagen, the predominant protein in mammals, is distinguished by its distinctive triple-helix configuration, offering exceptional tensile strength and stability (Brodsky et al., 1997; Di Lullo et al., 2002). Collagen’s isoelectric point varies according to its kind and source; however, it typically ranges from pH five to 6. There are several uses for collagen in the culinary, cosmetic, and pharmaceutical sectors (Thomas and Kelly, 1922; Lee et al., 2001; Avila Rodríguez et al., 2018). In addition to covalent cross-links that boost collagen’s mechanical strength, intra- and intermolecular hydrogen bonds preserve the protein’s three-dimensional structure. Collagen has a wide range of molar masses; Type I collagen has a molecular weight of about 300 kDa (Ramachandran et al., 1955; Bella et al., 1996; Gores et al., 2021). The growing demand for collagen and collagen-derived products in medical, cosmetic, and nutraceutical uses has resulted in a considerable global industry (Avila Rodríguez et al., 2018; Vieira et al., 2023).

Typically, gelatin is available in tablet, granule, or powder form and may require dissolution in water before utilization (Yang et al., 2016). The favorable attributes of this material, such as its ability to biodegrade, biocompatibility, and low toxicity, promote enhanced cell adhesion, differentiation, and proliferation. Simultaneously, it undergoes degradation by endogenous enzymes metalloproteinases within the body without eliciting an immunogenic reaction (C Echave et al., 2017; Echave et al., 2017; Echave et al., 2019). Gelatin is extensively utilized in various industries such as pharmaceuticals, food, cosmetics, and photography due to its unique functional properties. Gelatin is a food ingredient employed in the dairy, bread, beverage, and confectionery sectors to offer gelling, texturization, stability, and emulsification properties (Hanani et al., 2014). Gelatin is protein-rich and can be a viable alternative to fats and carbohydrates in some nutritionally balanced food products (Lv et al., 2019). Collagen, the predominant protein in both humans and animals, serves as the protein matrix for gelatin, providing a source of protein. Collagen is present in various body parts, with the highest concentrations found in bones, skin, tendons, and ligaments (Alipal et al., 2021). Gelatin is free of fat and cholesterol, making it a low-energy option. Additionally, it contains protective colloids that offer potential health benefits (Alipal et al., 2021). Pang and colleagues (Pang et al., 2014) suggest that gelatin exhibits significant emulsifying properties and has the potential to inhibit the coagulation of proteins from milk, soybean milk, and other sources in the presence of gastric acid within the stomach, thereby facilitating the process of food digestion (Pang et al., 2014).

## 2 Chitosan and gelatin processing techniques

Chitosan and gelatin can undergo diverse processing methods for various applications (Kou et al., 2021; Rigueto et al., 2022). Chitosan and gelatin are versatile materials that may be included in many products, including tablets, capsules, nano- and microparticles, beads, gels, and films. It can also be precipitated, blended, spray-dried, emulsified, and crosslinked (Bansal et al., 2011). Utilizing the solvent evaporation technique, membranes and films suitable for use in water and air filtering procedures may be produced. In the straightforward three-step solvent evaporation method, nano- or micron-sized biopolymer fillers are combined with polymer resin; occasionally, fibers are added to improve the mechanical qualities. The evaporation process is then started by pouring the combined solution into a glass container and heating it. The cast membrane or film can be removed from the container once it has evaporated (Alipal et al., 2021; Ilyas et al., 2022). The electrospun fibers are smaller in diameter and have a greater surface area. The potential differences produced between the spinneret and collector are applied to the polymer solution. The pendant-like droplets become jets due to the electric filling. At a critical point, the tension provided by the solution on the surface is exceeded by the electricity’s repulsion. This process causes fast whipping of the extruded polymer solution, which is unstable and causes nanofibers to develop on the collector due to evaporation (Sajkiewicz et al., 2014; Qasim et al., 2018; Green-Warren et al., 2022). During this procedure, biopolymer particles are blended with a polymer solution and maintained within a dispersion needle. The solution in the needle receives a high voltage. The droplets resist one another because of their equal charges. As a result of instability at the needle tip, the droplets begin to disperse into micron-sized particles and land on surfaces that are oppositely charged, all the while the solvent quickly evaporates (Choktaweesap et al., 2007; Islam et al., 2011; Sahoo et al., 2015; Garg et al., 2019; Hathout and Metwally, 2019). The best substitutes for chemically made wax coatings are biopolymers (Shyu et al., 2019; Shiekh et al., 2021). These coverings shield fruits from oxidation, reducing the amount of microorganisms present. To preserve their quality, fruits and vegetables are coated with gelatin and chitosan after harvest (Poverenov et al., 2014; Shyu et al., 2019).

## 3 Chitosan and gelatin as antimicrobial

The antimicrobial efficacy of chitosan is influenced by various fundamental factors, including the specific type of microorganism, the concentration and source of chitosan, its structural characteristics such as molecular weight and degree of acetylation, pH levels, environmental conditions, temperature, incorporation into composite materials, and the use of chitosan derivatives (Li and Zhuang, 2020; Yan et al., 2020). Chitosan with a lower molecular weight typically demonstrates greater antimicrobial efficacy due to its enhanced solubility, facilitating improved penetration of bacterial cell membranes (Gopi et al., 2020). Chitosan and chitooligosaccharide were incorporated into the cellulose matrix to enhance its antibacterial properties, with assessments conducted on both Gram-positive and Gram-negative bacterial strains. The findings demonstrated that Bacterial cellulose–chitosan and Bacterial cellulose–chitooligosaccharide composites displayed advantageous antibacterial properties compared to pure bacterial cellulose matrix. Additionally, these composites were characterized by reduced porosity and a compact structure. The composite material of bacterial cellulose/chitooligosaccharide demonstrates exceptional compatibility in food and medicinal contexts (Yin et al., 2020). Through the process of synthesis and testing, the antibacterial activity of chitosan NPs against tomato phytopathogens was evaluated. Pathogens include *Colletotrichum gelosporidies*, *Phytophthora capsici*, *Gibberella fugikuori*, *Sclerotinia sclerotiorum*, and *Fusarium oxysporum* are used in the testing procedure (Oh et al., 2019). Elwakil et al. (2020) prepared chitosan NPs and liposomes incorporating ethanolic cinnamon extract. They examined their chemical and physical characteristics before assessing their ability to heal wounds. Using chitosan and liposomes containing ethanolic cinnamon extract, they made a gel and tested it on diabetic mice. They discovered that the liposome/cinnamon gel was required for more effective treatment of bacterial infections and enzyme inhibition. Previous research has shown that chitosan exhibits greater efficacy in combating Gram-positive bacteria compared to Gram-negative bacteria. Additionally, chitosan has been found to possess inhibitory properties against various bacteria and fungi (Yan et al., 2021). The incorporation of chitosan and essential oil formulation in chitosan-derived edible packaging films enhanced the antimicrobial efficacy against a range of Gram-negative bacteria, notably *Escherichia coli*, *Pseudomonas aeruginosa*, *Klebsiella pneumoniae*, *Pseudomonas fluorescens*, *Shewanella baltica*, *Shewanella putrefaciens*, *Serratia* spp., as well as Gram-positive bacteria like *Staphylococcus saprophyticus* and *Staphylococcus aureus* (Altiok et al., 2010; Wang et al., 2011; Hafsa et al., 2016; Sani et al., 2019; Amor et al., 2021; Punia Bangar et al., 2021). Nevertheless, the growth of yeast, fungus, and mold is also suppressed (Pavlátková et al., 2023). Chitosan sheets were evaluated for their effectiveness against *Penicillium italicum* when combined with bergamot essential oil, demonstrating significant inhibitory properties. However, the inhibitory efficacy of the composite sheets was observed to diminish throughout the storage duration (Sánchez-González et al., 2010). The essential oils derived from cinnamon were found to have inhibitory effects on the growth of various fungi such as *Botrytis cinerea*, *Aspergillus oryzae*, *Penicillium digitatum*, *Aspergillus niger*, and *Rhizopus stolonifera* when applied to chitosan films (Mutlu-Ingok et al., 2020; El-araby et al., 2022). Li Z. et al. (2019a) noted that incorporating turmeric essential oil into chitosan led to notable anti-aflatoxigenic effects due to its demonstrated antifungal properties against *Aspergillus flavus*. Kavoosi et al. (2013) found that gelatin films containing thymol exhibited significant antibacterial efficacy, indicating their potential utility as antibacterial nano wound dressings for combating pathogens responsible for wound burns. This characteristic renders them appropriate for application as nano wound dressings with antibacterial properties for treating burns caused by pathogenic microorganisms (Ndlovu et al., 2021). They absorb wound exudates, maintain a moist wound environment, mimic the structure of the extracellular matrix (ECM), and exhibit antibacterial properties (Deng et al., 2022).

In the research conducted by Roy and Rhim (2021), it was observed that composite films comprising gelatin and chitosan exhibited effective antibacterial characteristics against pathogenic bacteria such as *E. coli* and *L. monocytogenes*. This antimicrobial efficacy was attributed to the inherent antimicrobial properties of chitosan. Furthermore, the study revealed that the antibacterial properties of the composite films were enhanced by the addition of functional fillers, cinnamon, and rutin (Roy and Rhim, 2021). Kurczewska (2022) states that gelatin and chitosan, along with their derivatives, are biodegradable polysaccharides characterized by biocompatibility, non-toxicity, and possessing antimicrobial and antifungal attributes (Kurczewska, 2022). The antibacterial efficacy of a chitosan-polyphenol extract was examined on Gram-negative bacterial strains including *Proteus mirabilis*, *P. aeruginosa*, *E. coli*, *Salmonella enterica*, *Salmonella typhimurium*, and *Proteus vulgaris*, demonstrating notable antimicrobial effects (Balti et al., 2017; Pires et al., 2022). Also, chitosan-polyphenol extracts exhibited notable antibacterial efficacy against Gram-positive bacterial strains including *Bacillus subtilis*, *Listeria innocua*, *Bacillus cereus*, *Streptococcus mutans*, *Listeria monocytogenes*, *S. aureus*, *Lactobacillus plantarum*, *Lactobacillus sakei*, and *Bacillus thuringiensis* (Balti et al., 2017; Zhang et al., 2019; Amankwaah et al., 2020; Zarandona et al., 2020). A hybrid hydrogel composed of chitosan and gelatin, supplemented with berberine hydrochloride (BBR) and gallic acid, demonstrated improved antibiofilm properties against *E. coli* and *S. aureus*. This formulation shows promise for potential use in biomedical settings (Liu et al., 2023). In addition, in a study, composite films made of chitosan, gelatin, and polyvinyl alcohol (PVA), which include antimicrobial agents such as *Duchesnea indica* extract, have demonstrated the ability to inhibit pathogens like *S. aureus*. This indicates the promising application of these films in food packaging (Choi et al., 2022).

## 4 Chitosan and gelatin as antioxidants

It is acknowledged that oxidation poses a significant challenge to the quality of food items. Additionally, the high-temperature processing of protein-based foods leads to the formation of heterocyclic amines, which are identified as carcinogenic compounds (Flores Llovera et al., 2019). Various elements, including processing parameters, culinary techniques, the existence of antioxidants, duration, and heat levels, can impact the generation of heterocyclic amines. Consequently, the mitigation or prevention of the creation of these carcinogenic compounds has emerged as a significant concern (Nadeem et al., 2021). A component that can postpone or prevent the oxidation of the molecules present in the medium is an antioxidant compound (Shahidi and Zhong, 2015). Gelatin and chitosan are both excellent options for creating antioxidant formulations and products since they both have antioxidant qualities (Jridi et al., 2014). The strong propensity of chitosan biopolymer to act as a hydrogen atom donor enhances its antioxidant capacity (Negm et al., 2020). Chitosan, when incorporated into food products as a food additive, can function as an antioxidant agent. This stops foods from forming heterocyclic amines (Liu T. et al., 2022b). Oz et al. (2017) investigated the effects of utilizing chitosan at varying concentrations of 0.25%, 0.50%, 0.75%, and 1% w/w on the quality of meatballs and the production of heterocyclic aromatic amines. The meatballs underwent preparation at different temperature levels (150°C, 200°C, and 250°C). Findings indicated that elevating the temperature from 150°C to 250°C resulted in a rise in the concentration of heterocyclic amine within the meatballs. However, upgrading the concentration of chitosan resulted in a notable reduction in the levels of the heterocyclic amine. Frozen meat’s 2-thiobarbituric acid reactive substance (TBARS) levels decreased by 70% when 1% chitosan was added. It has been observed that adding chitosan causes the free iron in beef heme proteins that are liberated during processing to be chelated (Tharanathan and Kittur, 2003). The antioxidant properties of chitosan have been found to correlate positively with its molecular weight, concentration, and viscosity. Chitosan derived from discarded crab shells was evaluated on herring tissue, and chitosan samples of varying viscosities were similarly assessed on fish specimens. The highest activity was observed with low-viscosity chitosan, and its action was similar to that of butylated hydroxytoluene (BHT), butylated hydroxy-anisole (BHA), and tert-butylhydroquinone (TBHQ). TBHQ, BHT, and BHA are synthetic antioxidants. Chitosan is believed to inhibit lipid oxidation in fish by sequestering ferrous ions (No et al., 2007; Muthu et al., 2021). Mirsadeghi et al. (2019) demonstrated that the incorporation of acid-soluble chitosan at a concentration of 1% into Huso fillets during the cooking process resulted in a significant decrease in the formation of heterocyclic amines, with an observed inhibitory effect of 68.09%. Adiletta and colleagues (Adiletta et al., 2019) assessed the functionality of enzymes including polyphenol oxidase, peroxidase, ascorbate peroxidase, and catalase to investigate the effects of chitosan-based coatings on preserving fig freshness. The findings indicated that applying a chitosan coating led to a notable enhancement in the levels of anthocyanins, flavonoids, and total polyphenols, as well as increased antioxidant activity in the preserved figs. This treatment also resulted in a decrease in oxidative stress and inhibited browning reactions when compared to the control group that did not receive the coating (Adiletta et al., 2019). The gelatin obtained from by-products of skipjack tuna (*Katsuwonus pelamis*) canning was refined to yield nineteen peptides exhibiting significant antioxidant properties. The gel’s exceptional clarity and strength are attributed to its elevated concentration of amino acids. These findings suggest that the antioxidant peptides derived from this gelatin could serve as potential supplements in health-promoting products to prevent ultraviolet-A damage (Zhang Y. et al., 2022b). In a study conducted by Sul et al., they found that adding carbon dots (CDs) from banana peel to chitosan/gelatin-based films improved the antioxidant properties of the films (Sul et al., 2023). Similarly, Roy and Rhim (2021) antioxidant activity of chitosan/gelatin-based films was evaluated using ABTS and DPPH methods. They have found that by adding cinnamon and rutin to chitosan/gelatin films, the antioxidant properties of the films increase. Furthermore, it has been shown various naturally derived bioactive compounds, including volatile oils, extracts from black or green tea, apple extract, purple and black eggplant, as well as purple and black rice, have been found to enhance the antioxidant properties of chitosan (Riaz et al., 2018; Yong et al., 2019a; Yong et al., 2019b; Aslı et al., 2020). This occurrence has been linked to the ability of the polyphenols present in the extract to eliminate free radicals through the release of phenolic hydrogen atoms (Pattnaik et al., 2022).

## 5 Chitosan and gelatin in food packaging

Over two hundred human illnesses, spanning from GI issues to cancer, are attributed to the consumption of food contaminated with pathogenic microorganisms, parasites, viruses, or toxic substances, resulting in approximately 600 million new cases and 420,000 fatalities annually (Suvarna et al., 2022). Food items derived from agricultural sources, such as fruits, are significantly tainted by harmful microorganisms due to inadequate safety measures (Paramithiotis et al., 2017). Similarly, the heightened levels of global trade have amplified the potential for disease transmission via contaminated food products, leading to an increased incidence of foodborne illnesses. Therefore, there is a necessity for intensified and targeted endeavors aimed at enhancing food packaging systems to mitigate the potential for foodborne illnesses (Bahrami et al., 2020). Food packaging systems provide a range of advantages, including improved handling, extended shelf life, and safeguarding against physicochemical harm during storage and transportation. As such, they play a vital function within the worldwide food sector. Additionally, individuals are seeking food packaging materials that are innovative, cost-efficient, environmentally sustainable, and effective in ensuring the safety, nutritional value, and quality of products (Omerović et al., 2021). Novel packaging techniques incorporating antioxidant and antibacterial properties are currently under development to enhance food safety measures (Roy and Rhim, 2021). Biopolymers, including proteins and carbohydrates, offer numerous advantages over traditional synthetic polymers when utilized as a stable matrix in active packaging films (Atarés and Chiralt, 2016). Using petroleum-based materials has negative environmental implications since they are neither renewable, recyclable, reused, or obtained responsibly (Cruz et al., 2022; Zhao et al., 2022). In a study, Sul et al. (2023) prepared active films with an equal mixture of chitosan and gelatin and added CDs from banana peel to be used for food packaging. They discovered that adding CD to chitosan/gelatin functional films has a wide range of applications in food packaging, particularly in extending the shelf life of meat and preserving its visual quality. Hamann et al. (2022) prepared active films consisting of 15% gelatin, 1% green tea extract, and 30% glycerol. These films were incorporated into the outer layer of the newly prepared sausages. The results of their study indicated that the application of an active gelatin film on sausages led to a decrease in TBARS levels during refrigerated storage. Ultimately, they determined that gelatin films incorporating green tea extract show potential as a viable alternative for prolonging the shelf life of sausages (Hamann et al., 2022). Wu et al. (2019) developed chitosan films by incorporating curcumin-loaded mesoporous silica nanoparticles (CMSNP) through the solvent-casting technique. The film’s dimensions, mechanical properties, and water vapor permeability were determined to be 0.0931 ± 0.0021 mm in thickness, 19.87 ± 1.02 MPa in tensile strength, 25.46% ± 2.16% in elongation at break, and 15.21 ± 1.83 g 10–11/s m^2^ Pa in water vapor permeability. The CMSNP film and the plain Chitosan/Curcumin blend film demonstrated zones of inhibitions (ZOI) measuring approximately 7.5 and 8 mm against *E. coli* and 8 mm and 10 mm against *S. aureus*, respectively (Wu et al., 2019). Siripatrawan and Kaewklin (2018) created active packaging using chitosan and Titanium dioxide (TiO2) NPs at varying concentrations (0%, 0.25%, 0.5%, 1%, and 2% w/w). The chitosan film with a 1% concentration of TiO2 NPs exhibited antibacterial effects against various strains of bacteria, including *S. aureus*, *E. coli*, *P. aeruginosa*, and *S. typhimurium*, as well as fungi such as *Penicillium* and *Aspergillus*. Therefore, the findings indicated that chitosan-TiO2 nanocomposite films have the potential to serve as effective active packaging materials (Siripatrawan and Kaewklin, 2018). Dehghani et al. (2022) developed coating dispersions using combinations of fish gelatin, conjugates, and bitter almond gum in varying ratios of 1:2, 2:1, and 1:1. The researchers examined the impact of coating suspensions on the physicochemical and qualitative characteristics of tomatoes during a 28-day storage period at a temperature of 20°C. The researchers discovered that combining fish gelatin with a greater proportion of bitter almond gum has the potential to be an effective method for creating coating dispersion and preserving the quality of fruits over time (Dehghani et al., 2022). Wang et al. (2018) utilized a blend of chitosan and gold NPs to demonstrate the frozen state and temperature profile of food by observing the color variation resulting from the aggregation of gold NPs due to their localized surface plasmon resonance. Moreover, due to the alterations in the physical and chemical properties of food, chitosan-derived materials developed for tracking pH fluctuations in food products can also detect bacterial presence and oxidative degradation of food. Singh et al. (2021) incorporated gallic acid and sodium carbonate into the chitosan film to create an oxygen-absorbing substance. The findings indicated a reduction in the mechanical properties of the chitosan films with escalating levels of sodium carbonate and gallic acid additives. This phenomenon could be attributed to the significant quantity of sodium carbonate disrupting the internal structure of the chitosan film. El-Gioushy et al. (2022) conducted research on the utilization of nano-chitosan as a functional edible coating film at varying concentrations (1, 2, and 3 cm^3^/L) to improve the shelf life and quality attributes of Barhi cultivar date palm fruits during refrigerated storage at ±2°C for 70 days. Their findings indicated that applying three cm3/L of nano-chitosan as a spray on Barhi date fruits yielded optimal outcomes after the storage period (El-Gioushy et al., 2022). In an investigation involving gelatin-chitosan and pectin-chitosan, films and coatings were produced. The researchers integrated lemongrass essential oil, Zn, or ZnO as active components into the films. The thermal analysis indicated a notable level of stability. Regarding mechanical properties, the gelatin-chitosan films exhibited favorable attributes suitable for practical utilization. Furthermore, the antibacterial efficacy was evaluated, revealing a synergistic interaction among the active components integrated within the films. The unique aspect of this research lies in the experimentation conducted on the protective coating applied to containers holding raspberries. The most favorable microbiological performance was observed in containers treated with a gelatin-chitosan emulsion containing ZnO. The longevity of the fruit was extended by all formulations examined, ranging from four to 8 days (Jovanović et al., 2021). Table 2 Summary of recent studies on the use of chitosan/gelatin composites reinforced for food packaging.

**TABLE 2:** Recent studies on the use of chitosan/gelatin for food packaging.

Biodegradable matrix	Reinforcement	Conclusion	Reference
Gelatin/chitosan	Curcumin	Based on the results, protein-rich animal goods like meat and seafood are protected, and their freshness is tracked using gelatin, chitosan, and curcumin nanofiber packing, which has a lot of promise	Duan et al. (2023)
Chitosan	Nanocellulose	While maintaining water vapor permeability, adding nanocellulose increased the material’s thermal stability and oxygen barrier. The chitosan/nanocellulose films’ increased tensile strength and Young’s modulus indicated improved mechanical characteristics. These films showed fungicidal action against *Candida albicans* as well as bactericidal impact against both Gram-positive and Gram-negative bacteria	Costa et al. (2021)
Chitosan/gelatin	Green synthesized zinc oxide (ZnO)	-The hybrid films reinforced with ZnO NPs had better compactness, elongation-at-break, and thermal stability -The produced hybrid nanocomposite films can be used as a biodegradable substitute for fresh fruit and vegetable postharvest packaging	Kumar et al. (2020b)
Chitosan	Peppermint essential oil and berberis extract	The turkey breast meat treated with a chitosan solution containing berberis extract and peppermint essential oil showed significantly reduced bacterial counts and oxidation levels under refrigeration	Yuceer and Caner (2014)
Chitosan/gelatin	AgNPs	The produced composite films were considered a biodegradable and biocompatible food packaging material and a substitute for petroleum-based plastics since they had all the necessary qualities for packaging material, including mechanical strength, flexibility, barrier properties, and antimicrobial activity, according to the results	Ediyilyam et al. (2021)
Chitosan/gelatin	Apple Peel NPs	The outcomes showed that the films made of chitosan/gelatin and apple peel extract had strong antioxidant qualities, suggesting they could be developed as a bio-nanocomposite food packaging material for the food sector	Riaz et al. (2020)

The capacity of food packaging materials to regulate the transfer of water vapor is one of the most critical factors. This characteristic, which stops moisture loss and oxidation, is essential for preserving the quality and lengthening the shelf life of perishable foods. It has been discovered that chitosan films have a low water vapor transmission rate, which helps to keep food items wet. Because chitosan is hydrophilic, it may create strong hydrogen bonds that aid in forming a barrier against water vapor (Cazón et al., 2019; Long et al., 2023). Gelatin exhibits notable gas barrier characteristics and swelling behavior in aqueous environments, yet it is hindered by limited mechanical strength and susceptibility to water vapor permeation (Sobral et al., 2001; Zhang et al., 2010). Pellá et al. (2020) developed a thin film with low solubility and exceptionally low water vapor permeability by physically mixing cassava starch, gelatin, casein, and sorbitol. The fruit with coating saw less quality loss and had a higher concentration of soluble solid and vitamin C due to the delayed rate of chlorophyll breakdown. In addition, the guava fruit covered with this film had a 2-day shelf life increase. After 9 days of storage, fruit with a coating remained green, but fruit without a coating had turned brown after 3 days. It is evident that gelatin-based mixtures efficiently postpone the ripening and rotting of fruit (Pellá et al., 2020). A combination coating consisting of chitosan, gelatin, gallic acid, and clove oil was created by Xiong et al. (2021) to study the fresh-keeping properties of fresh salmon fillet that was kept in a refrigerator for 15 days at 4°C. It was found that the combination coating may successfully stop the salmon fillet’s brightness from decreasing. This may be because the film has an antioxidant effect that protects and isolates the fish from the environment. Adding chitosan/gelatin and clove oil significantly enhanced the antioxidant and antibacterial effects, resulting in a shelf-life extension of at least 5 days. Additionally, the gas and water vapor permeability of the gelatin coating and the PH value of all the coated fillet samples decreased (Xiong et al., 2021).

The studies summarized in Table 2 collectively highlight the potential of chitosan/gelatin composites reinforced with various nanomaterials for food packaging applications (Yuceer and Caner, 2014; Kumar et al., 2020b; Riaz et al., 2020; Costa et al., 2021; Ediyilyam et al., 2021; Duan et al., 2023). The reinforcement materials consistently increase the mechanical strength, barrier characteristics, and antimicrobial activity of the composites. However, the environmental impact and cost-effectiveness of large-scale production of these composites need to be investigated further. Future research should focus on addressing these gaps and developing standardized testing protocols.

### 5.1 The process of producing food packaging films

#### 5.1.1 Casting method

The casting method, solvent casting, is the predominant technique utilized for film formation in laboratory and pilot-scale settings. The procedure for preparing biopolymer films includes dissolving the biopolymer in an appropriate solvent, pouring the solution into a mold, and subsequently drying the casted solution. The initial stage involves choosing the polymer or combination of polymers to form the fundamental film. The selected polymer is dissolved in an appropriate solvent, a critical step as the film-forming capability primarily hinges on the solubility of the polymer rather than its melting characteristics. During the casting process, the solution obtained is poured into a preselected mold or a Petri dish coated with Teflon. During the drying phase, the solvent undergoes evaporation, leading to the formation of a polymer film that attaches to the mold. Various types of air dryers, including hot air ovens, microwaves, tray dryers, and vacuum driers, are employed to efficiently eliminate solvents and facilitate the successful peeling of formed films (Jensen et al., 2015; Suhag et al., 2020). To increase chitosan’s solubility, dissolves in an acidic solution like acetic acid. Water is used to dissolve gelatin, and heat is frequently used to promote total dissolving (Park et al., 2002; Chuaynukul et al., 2018). Usually, the casting procedure is carried out at ambient temperature (20°C–25°C). To preserve their fluidity, gelatin solutions can be cast at somewhat higher temperatures (30°C–40°C) (Acierno et al., 1999; Aniunoh et al., 2006; Biscarat et al., 2015). It might be necessary to wash chitosan films with distilled water or a neutralizing solution such as sodium hydroxide to eliminate any remaining solvent and balance the pH (Kim et al., 2006; Chang et al., 2019).

#### 5.1.2 Extrusion method

The extrusion technique is commonly employed in polymer processing for producing polymeric films. This procedure modifies the composition of the substances and enhances the physical and chemical properties of the extruded material. The extrusion process typically consists of three main stages: (i) feeding, (ii) kneading, and (iii) heating as the exits the machine. Initially, the film-forming mixture is introduced into the feeding zone and compacted with air assistance. This procedure is commonly called a dry process due to its limited use of water or solvents. Plasticizers like sorbitol or polyethylene glycol are employed in concentrations ranging from 10% to 60% by weight to enhance the flexibility of the film. During the kneading process, there is an elevation in the strain, temperature, and density of the mixture. Ultimately, during the heating phase, the thermal energy fluctuates within the temperature range of 120°C–170°C. This procedure relies on the thermoplastic nature of polymers, which occurs when plasticization and heating occur above the glass transition temperature under low moisture conditions (Fitch-Vargas et al., 2016; Suhag et al., 2020). The extrusion method is indeed utilized for producing films of chitosan and gelatin. This method offers advantages such as continuous production, uniform film thickness, and scalability for industrial applications. However, specific considerations must be made due to the thermal sensitivity and properties of chitosan and gelatin. High temperatures have the potential to degrade both gelatin and chitosan. For these biopolymers, the 120°C–170°C temperature range usually employed for polymer extrusion may be too high (Aider, 2010; Pelissari et al., 2011; Hanani et al., 2012; Krishna et al., 2012).

#### 5.1.3 Electrospinning method

**FIGURE 3:**
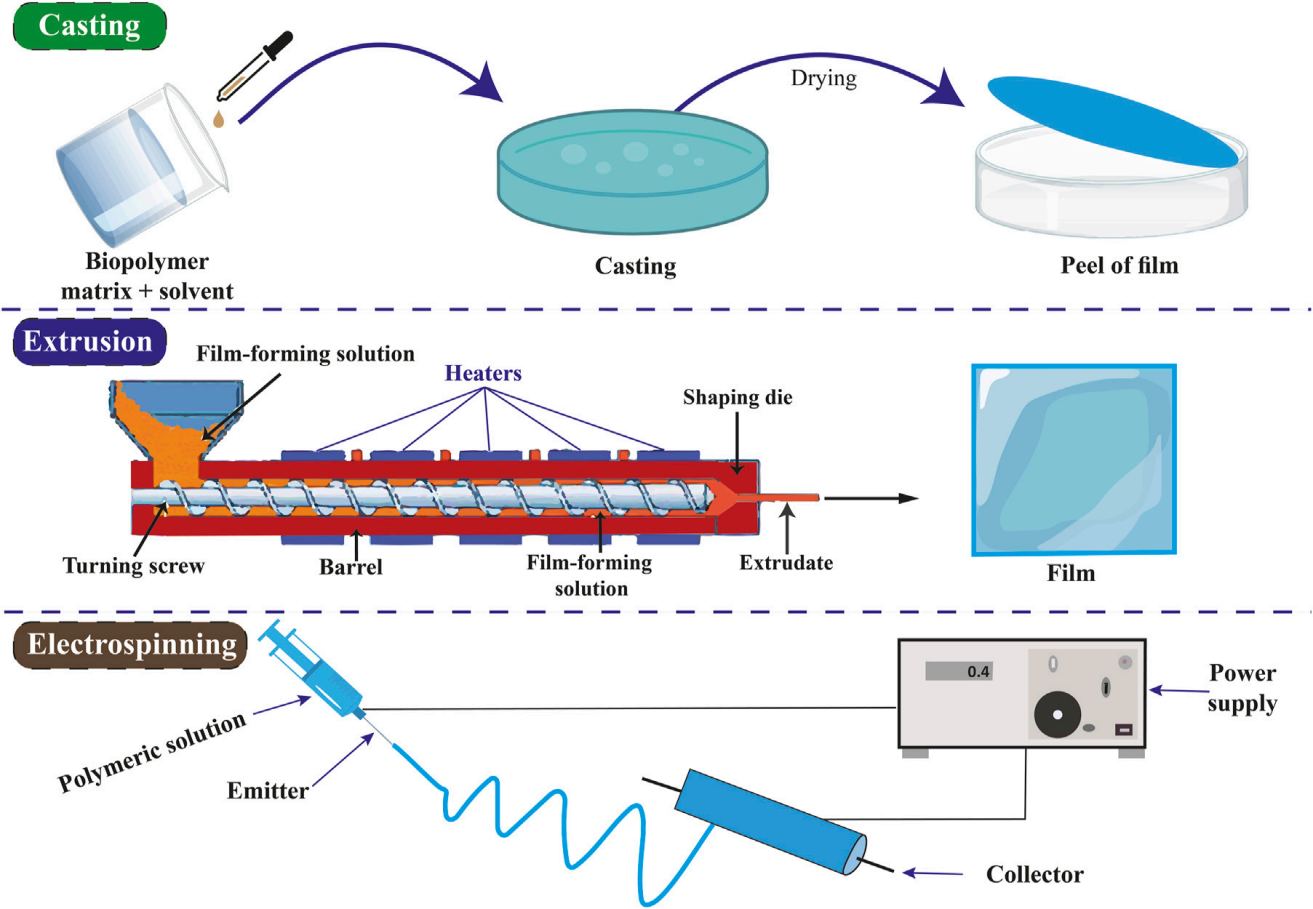
Methods of preparation of packaging films.

The electrospinning technique produces a nonwoven mesh composed of micro- or nanofibers. This method involves the application of high-voltage electricity to a liquid solution and a collector, resulting in the extrusion of the solution from a nozzle to form a jet. During the drying phase, the fibers generated by the jet are accumulated on the collector. Electrospinning represents a rapid and efficient method for producing micro- or nanoscale polymer fibers. The presence of polymers in the electrospinning solution results in modifications to its viscosity, molecular weight, surface tension, conductivity, concentration, solvent, and various other characteristics, all of which play a crucial role in influencing the electrospinning process. During the electrospinning process, the dispersed fibers undergo self-assembly due to electric charges, which are controlled by mechanical forces and geometric conditions. Electrospinning was employed to fabricate nanofiber polymers, including chitosan, collagen, alginate, cellulose, polyesters, and polyurethanes (Nayak et al., 2012; Yang et al., 2020). The methods of preparing packaging films are shown in Figure 3.

To get the required viscosity for electrospinning, gelatin is usually dissolved in acetic acid-containing aqueous solutions or in a mixture of water and ethanol. Concentrations typically vary from 10% to 20% w/v, contingent upon the gelatin’s molecular weight. Typically, chitosan is dissolved in 1%–2% v/v diluted acetic acid solutions to get the necessary electrospinnable viscosity (Ohkawa et al., 2004; Choktaweesap et al., 2007; Habibi and Hajinasrollah, 2018).

## 6 Chitosan and gelatin in microencapsulation technology

Microencapsulation is a novel technological advancement that involves safeguarding diverse food components or functional constituents from different processing conditions by encapsulating them within a polymeric or nonpolymeric substance. This encapsulation method enables the controlled release of these components under specific circumstances. Furthermore, it improves the sensory attributes of food products by concealing undesirable tastes, aromas, and flavors while bolstering food safety by suppressing microbial growth (Choudhury et al., 2021). The effectiveness of the capsule is contingent upon the characteristics of both the wall and base materials. Significant outcomes can be achieved by utilizing a blend of the wall material to formulate the microcapsules. The primary active constituents in Turkish oregano extract, carvacrol and rosmarinic acid, have demonstrated enhanced release in laboratory settings when encapsulated with gelatin, Tween 20, gum arabic, and cyclodextrin as coating agents (Baranauskaite et al., 2019). Chitosan possesses characteristics that render it a suitable material for encapsulating a diverse range of bioactive compounds. This characteristic renders it valuable across various sectors, including food, biomedical, pharmaceutical, agricultural, environmental, and industrial (Ho et al., 2021). This polymer is utilized to encapsulates multiple substances such as food ingredients, medications, vitamins, lipids, essential oils, vaccines, hemoglobin, and microbial metabolites (Raza et al., 2020). Chitosan and its encapsulated derivatives are extensively employed in agriculture with various environmentally friendly products, including biopesticides, organic fertilizers, seed treatments, soil conditioners, and growth-promoting agents (Ambaye et al., 2022). Chitosan has been employed as a co-encapsulation agent for curcumin and resveratrol in various studies (Chen et al., 2020). Chitosan is used in the creation of nanocomposite active substances incorporated into films to suppress the proliferation of fungi like *A. niger*, *Aspergillus parasiticus*, *A. flavus*, and *Penicillium chrysogenum*, thereby facilitating the management and suppression of these harmful microorganisms (Hossain et al., 2019). Non-toxic chitosan has been extensively employed for the encapsulation of anthocyanins. Anthocyanin-chitosan NPs are created through the establishment of non-covalent interactions, such as weak ionic bonding and hydrogen bonding (Sreerekha et al., 2021). According to the findings, applying a dual coating comprising chitosan and a polyanionic polysaccharide to stabilize anthocyanins resulted in a notable increase in encapsulation efficiency. This approach also conferred resistance against auto-oxidation, ascorbic acid degradation, exposure to heat, and neutral environmental conditions (Tan et al., 2021). The covalent bonds established between proteins and polysaccharides play a crucial role in increasing stability and preventing the release of anthocyanins in challenging conditions (Gonçalves et al., 2018). Overall, gelatin and chitosan show promise as polymeric matrices for microencapsulation.

The following specific techniques are commonly used for producing gelatin and chitosan particles.

### 6.1 Emulsification-solvent evaporation method

Using this procedure, a polymer solution is created by dissolving biopolymers in an organic solvent (such as dichloromethane or ethyl acetate). This solution is used to disperse or dissolve the active component, creating an emulsion (water-in-oil or oil-in-water). After that, the emulsion is agitated to allow the solvent to evaporate and solid particles containing the active component to form (Watts et al., 1990; Yang et al., 2001; Essa et al., 2020).

#### 6.1.1 Ionic gelation method

Chitosan is well-suited for this approach because of its polycationic properties. The process entails the interaction of chitosan with an anionic polymer or cross-linker (such as sodium tripolyphosphate or alginate) to create NPs or microspheres (Fan et al., 2012; Desai, 2016).

#### 6.1.2 Coacervation method

This procedure, which includes phase separation, frequently uses gelatin. Warm water dissolves the gelatin, and the active component is then mixed into the mixture. To cause phase separation, an appropriate coacervating agent (such as gum arabic or gelatinized starch) is applied (Veis and Aranyi, 1960; Burgess and Carless, 1985).

#### 6.1.3 Spray drying

Using this method, the active component and a solution or suspension of gelatin or chitosan are atomized and placed into a heated chamber to dry quickly. Microcapsules of the encapsulated item are left behind as the solvent swiftly evaporates (Bruschi et al., 2003; Estevinho et al., 2013).

## 7 Chitosan and gelatin in water treatment

Water serves as the fundamental cornerstone for supporting and sustaining life on the planet Earth. Access to clean drinking water is considered a basic human right; however, the issue of water scarcity has become a significant global challenge in contemporary times. This is primarily attributed to the swift expansion of industries, agriculture, and technology, driven by the increasing world population. This phenomenon has resulted in the excessive utilization and pollution of current freshwater reservoirs (Kumar et al., 2014; Zhang et al., 2016a). According to a report published by the World Health Organization, the implementation of fundamental water hygiene and sanitation practices has the potential to reduce waterborne illnesses such as diarrhea by 35% (Pendergast et al., 2011; Sen Gupta et al., 2023). Inadequate sanitation can result in the introduction of new pathogens like the Ebola virus and the more recent SARS-CoV-2 into water systems, thereby presenting potential health risks for individuals (Quilliam et al., 2020; Lahrich et al., 2021). Two-thirds of the world’s population is predicted to live in water-stressed areas with intermittent or persistent freshwater shortages by 2050, according to another analysis (Lee et al., 2016; Samantaray et al., 2018). In almost every facet of human existence, including sanitation, industry, agriculture, power production, building, and transportation, freshwater is required (Chelu et al., 2023). Even in minute concentrations, heavy metals including lead, nickel, copper, cadmium, zinc, mercury, arsenic, chromium, bismuth, cobalt, and iron are detrimental to the environment and public health (Engwa et al., 2019). It is crucial to remove numerous dangerous pollutants from wastewater, including paints, heavy metals, medicines, healthcare products, detergents, derivatives, and industrial by-products, since they not only contaminate water but also pose a toxic risk to the ecosystem (Ethaib and Zubaidi, 2022; Chelu et al., 2023). The GelYst biosorbent, used to enhance the extraction and biosorption of Cr (VI) from water, is made of yeast and gelatin. The use of this biosorbent in the treatment of water has proven effective (Mahmoud, 2015). Because of their numerous natural qualities, hydrogels based on natural polysaccharides are now employed as coagulants and adsorbents in the filtration of drinking water (Chelu et al., 2023). Chitosan-based hydrogels are promising matrices for treating contaminated waters because of their low cost, excellent chemical stability, mechanical solid and heat resistance, and ease of recovery—reusing the hydrogel and the contaminants (Chelu et al., 2023). Moreover, the mechanical strength of hydrogels based on chitosan can be increased by crosslinking with synthetic or biopolymers or by incorporating NPs. Through hydrogen bonding and electrostatic interactions, chitosan may readily adsorb various contaminants (such as heavy metals and dyes) because of its abundance of hydroxyl and amino groups (Luo et al., 2022). Chitosan has drawn a lot of interest in water treatment applications because of its unique qualities, which include cationic, high adsorption capacity, macromolecular structure, low cost, and abundance as compared to other commercial adsorbents (Ahmed et al., 2020). It has been claimed that chitosan or different variations of this biopolymer may successfully remove various metals and other contaminants (Russo et al., 2021). Furthermore, a gelatin/chitosan composite has been created and applied to pesticide wastewater samples, demonstrating a high level of atrazine and fenitrothion removal effectiveness (Attallah et al., 2021).

## 8 Chitosan and gelatin in drug delivery

Since drug distribution affects a therapeutic agent’s safety and effectiveness, it is a crucial component of modern medicine. Targeted medication distribution is a challenging process that has to get past several obstacles, such as the liver and kidneys’ capacity to eliminate drugs, the bloodstream’s quick breakdown, and biological membranes’ limited permeability. A suitable carrier or vehicle is required to protect the drug from degradation, prolong its circulation, and improve its localization at the target site. The optimum drug delivery vehicle should have numerous critical traits, such as biodegradability, biocompatibility, and controlled release properties (Homayun et al., 2019). Biocompatibility assures that the delivery vehicle has no adverse effects on the body. In contrast, biodegradability ensures that it may be safely metabolized and eliminated from the body once its job is complete. Drugs with controlled release qualities release the medication in a regulated way over a predetermined time, maintaining therapeutic levels and minimizing dose frequency (Kantak and Bharate, 2022). The distinct attributes of gelatin and chitosan make them popular choices for medication delivery applications (Sethi and Kaith, 2022). Chitosan is authorized for use in tissue engineering and medication delivery applications by the FDA and is categorized as “Generally Recognized as Safe” (GRAS) (Kantak and Bharate, 2022). According to the results of several acute toxicity tests, chitosan has an LD50 of more than 16 g/kg when given orally to mice, and it is safe to use throughout the body (Kean and Thanou, 2010). One of chitosan’s main advantages is its adaptability; it can be made into various dosage forms, each with unique qualities and uses (Desai et al., 2023). According to research on rabbit eyes by Zhang et al. (2016b), chitosan does not irritate the eyes. Santhi et al. (2017) employed spontaneous emulsification and a cross-linking strategy to generate fluconazole-loaded chitosan NPs. Using the cup-plate approach, they evaluated these NPs’ antifungal properties in comparison to those of conventional eye drops. The average size of these particles was 152.85 ± 13.7 nm. It was found that every drug-loaded NP had an ideal (50%) drug-loading capacity. After their investigation, they concluded that the fluconazole-formulated chitosan NPs were a functional drug loading, antifungal efficacious, and delayed release delivery method for fluconazole (Santhi et al., 2017). Gelatin capsules can regulate medicine dosage, effectively increase drug use, and improve drug consumption and storage convenience (Gullapalli et al., 2017). Gelatin readily binds to medication molecules due to its high water solubility (Yildirim et al., 2023). Ramanathan et al. (2022) released 5-fluorouracil (5-FU), which is very hydrophilic, and methotrexate (MT), which is less hydrophilic, using polyhydroxy butyric acid/gelatin nanofibers. When the release of two distinct drug classes with varying hydrophilic characteristics was evaluated, the highly hydrophilic 5-FU was released first and at a faster pace than MT. MT and 5-FU showed a steady release rate after 24–96 h, which may efficiently satisfy the requirements of various medications in the postoperative management of cancer (Ramanathan et al., 2022). Furthermore, by increasing hydrophilicity, gelatin may alter other anticancer drug delivery vehicles, improving the stability *in vivo*. Li et al. (2022) created a gelatin, chitosan, and doxorubicin NP by loading doxorubicin (DOX) into chitosan and utilizing gelatin to crosslink it. When compared to chitosan/DOX NPs, gelatin, which functions as the nanoshell of the NPs, efficiently enhances the loading of DOX and exhibits high stability *in vivo*. It can also speed up how cancer cells absorb drugs (Li et al., 2022). Similarly, by hydrophobic interactions on the graphene surface, gelatin may also be employed to improve the durability of graphene structures *in vivo* (Hasanin et al., 2022). Many pharmaceuticals have been found to work better when chitosan is added. Using chitosan as the polymer, rifampicin, an antitubercular medication, was developed as a powdered dry NP inhalation. This structure showed that the medication may be released continuously for up to 24 h without endangering any cells or organs (Rawal et al., 2017). Debnath et al. (2018) administered prothionamide, an antitubercular drug, via the lungs as chitosan-coated NPs. The drug’s inhalation half-life in the lungs was extended by this modification. The antifungal medicine itraconazole has limited oral solubility; hence, Jafarinejad et al. (2012) created chitosan NPs for the antifungal agent’s pulmonary administration in a dry powder format. By including chitosan NPs, lactose, mannitol, and leucine in the formulation, they improved the drug’s aerosolization properties. There was a consequent rise in the pulmonary deposition of itraconazole. Table 3 provides an overview of many popular chitosan/gelatin-based delivery methods.

**TABLE 3:** Chitosan/gelatin-based systems for biomedical and pharmaceutical applications.

Type of system	Overview	Method of preparation	Key attributes/Features	Reference
Tablets	To regulate medication release, enhance stability and shelf life, and improve the mechanical qualities of tablets, they are utilized as a matrix material during tablet manufacture	- Wet granulation - Direct compression	- Ability to produce oral mucoadhesive pills - Extends the profile of medication release - increases the gastric stability of medications taken orally -A smooth, appealing coating that made swallowing the pills easy and appealing to the user	Badwan et al. (2015), Al-Nimry et al. (2021)
Microspheres	The particles are round and range in diameter from 10 μm to 1,000 μm. Variants that enable modification of the release profile include hollow, core-shell, and fibrous microspheres	-Spray drying -Coacervation/precipitation -Ionotropic gelation -Emulsion or thermal cross-linking	-High effectiveness of trapping and drug loading -Sustained drug release -Adaptability in the administration’s path -Stabilizes trapped biomolecules physically	Esposito et al. (1996), Saranya et al. (2018)
NPs	Because of their variable size (1–100 nm) and capacity for surface modification, these particulate systems are used as flexible platforms for the targeted administration of medications, DNA, and proteins	-Cross-linking and emulsification -Desolvation technique -Polyelectrolyte complexation -Modified ionic gelation -Reverse micellization -Emulsion-Solvent Evaporation	-High site-specific medication localization (in cancer) by improved permeability and retention effect or by using targeting ligands -Adaptable to produce stimuli-triggered medication release (pH, redox, temperature) -Enables the co-delivery of pharmaceutical compounds	Sahoo et al. (2015), Garg et al. (2019)
Nanofibers	They are a unique platform in which the medication is linked to or enclosed in fibers that have nanometer-sized dimensions. Nanofibers’ high surface area to volume ratio qualifies them for regenerative medicine and controlled drug release applications	-Templating -Melt/solution blowing -Electrospinning	-Their large surface area about their volume enables effective release and substantial drug loading -By changing the concentration and composition of polymeric materials, drug release may be modified -Bilayered or trilayered nanofibers can be manufactured to combine several medicines	Al-Jbour et al. (2019), Arun et al. (2021)
Hydrogels	These are chains of cross-linked polymers that come together to form a 3D network that can hold a lot of water. Hydrogels with specific physicochemical qualities for various biomedical applications can be made more accessible by the molecular control of the gelation chemistry involved	-Physical and chemical crosslinking -Enzymatic crosslinking -Photo-crosslinking	-Excellent biocompatibility, biodegradability, and injectability -It is possible to regulate the degradation of the platform better and, therefore, the rates of drug release by making molecular-level alterations -It is possible to include *in situ* forming qualities -Has a hydrating effect when used in tropical climates	Peers et al. (2020), Akın Şahbaz (2023)
Membranes	They are pliable, thin sheets that may be formed to precise measurements and serve as a dose form. They also make it easier for medications to be released directly into biological settings	- Hot pressing - Solvent casting - co-electrospinning	- Chitosan enhances the way polar medications are transported across epithelial surfaces - Chitosan is a polymer that has the potential to bind cells and attract negatively charged cell surfaces because of its cationic polyelectrolyte structure	Al-Baadani et al. (2021), Trombino et al. (2021)
Microgranules/powder	These are subclasses of solid dosage forms made of gelatin and polymeric chitosan combined with non-uniform micron-sized drug particles	- Salt-/Organic solvent-induced precipitation - Gelation - Spray drying	- Simpleness of usage, management, and preparation - Enhances the chemical stability of integrated medications - Powders and granules with small particle sizes dissolve quickly in the body, improving their bioavailability - Useful for large-dose, bulky medications	Singh et al. (2012)

## 9 Chitosan and gelatin in tissue engineering

**FIGURE 4:**
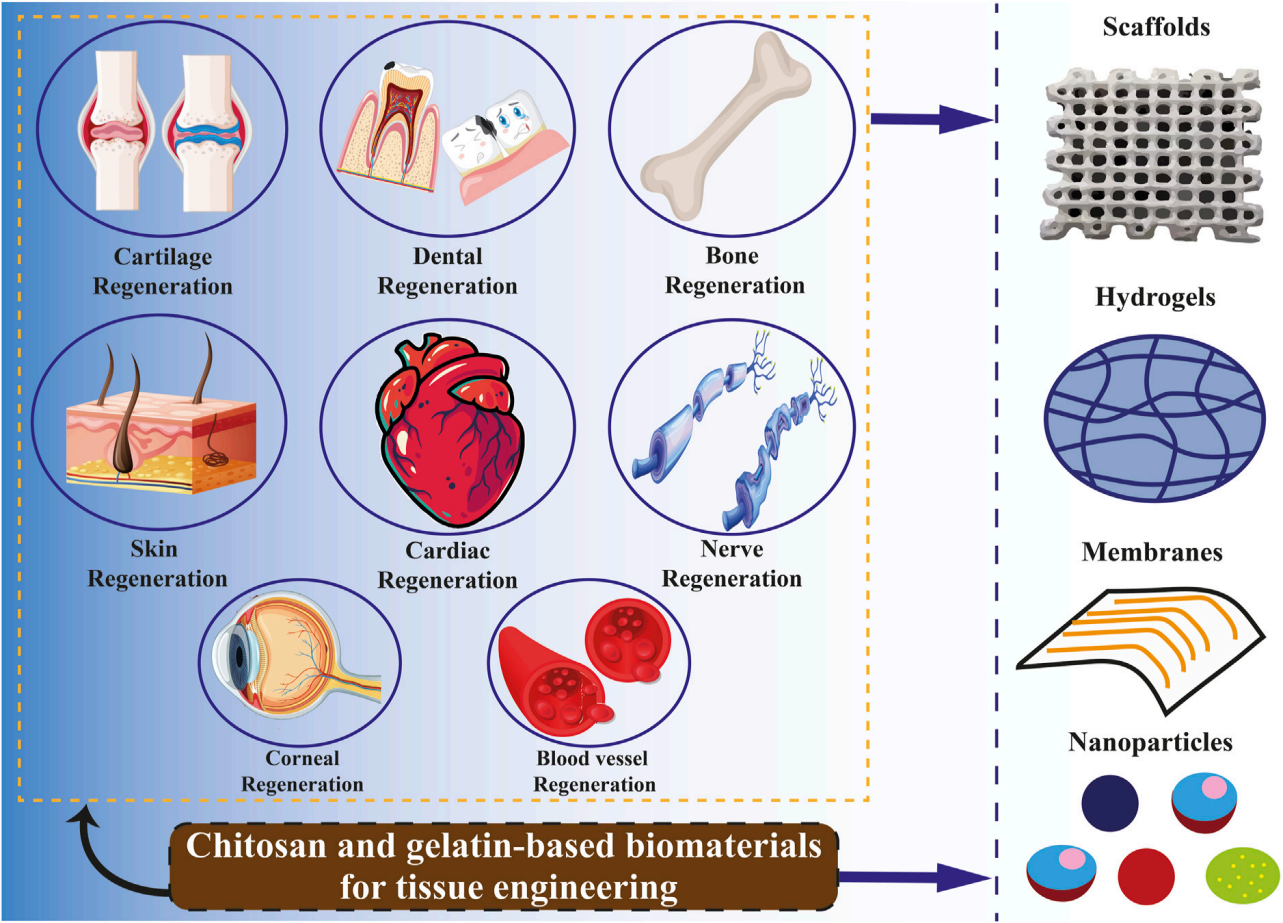
Use of chitosan and gelatin biomaterials for tissue regeneration.

Tissue engineering is an emerging field of study that integrates technologies from various research disciplines, such as biology, engineering, medicine, chemistry, material science, and pharmacy (Biswal, 2021). In light of the present health concern of organ and tissue failure, this interdisciplinary field may offer a medicinal substitute. According to recent reports from the United States (US) government, 107,000 individuals are waiting for organ transplants, and up to 17 people on these lists pass away every day. In Europe, six new patients are added to the waiting list every hour, with an estimated 18 individuals succumbing daily while awaiting medical care (Lukin et al., 2022). In this line, tissue engineering is to advance the utilization of biomaterials, such as scaffolds, in facilitating effective tissue regeneration and restoration. These are formations characterized by pores of diverse sizes and shapes, which may be interconnected or isolated. These attributes are determined based on the specific cell type of the tissue or organ in which a scaffold is intended to be utilized (Naghieh et al., 2018; Rahmani Del Bakhshayesh et al., 2018; Rider et al., 2018). Natural polymers such as gelatin, collagen, and chitosan are favored in tissue engineering due to their low antigenicity, ability to degrade naturally, compatibility with biological systems, and resemblance to the standard ECM Figure 4 (Khalilimofrad et al., 2023). Utilizing a blend of polysaccharide and protein, exemplified by chitosan and gelatin, has demonstrated efficacy as a viable approach in emulating the characteristics of the native ECM. Consequently, this combination is a promising alternative for fabricating scaffolds intended for tissue engineering applications (Cheng et al., 2014; Rosellini et al., 2018; Ghaee et al., 2019). The limited mechanical strength of biomaterials derived from pure chitosan restricts their potential applications. Hence, chitosan has been frequently combined with other polymers to leverage their synergistic properties. Chitosan can readily be integrated into a hybrid composite material with various natural polymers like gelatin, silk, DNA, cellulose, proteins, and wool, owing to the presence of hydroxyl and amino groups, which facilitate compatibility and interaction between the components (Khalilimofrad et al., 2023). Combining chitosan with gelatin is a successful approach due to an Arg–Gly–Asp (RGD)-like sequence in the protein, which enhances cell adhesion and migration. This interaction results in the formation of a polyelectrolyte complex with the polysaccharide (Huang et al., 2005; Thein-Han et al., 2009; Kumar et al., 2017; Rajasree et al., 2020). Xu et al. (2021b) created scaffolds utilizing gelatin, chitosan. They decellularized ECM through freeze-drying, demonstrating notable biocompatibility, effective antibacterial properties, and suitable mechanical characteristics conducive to applications in skin tissue engineering. Zhang et al. (2020) constructed sandwich-like scaffolds using polycaprolactone, gelatin, and chitosan through electrospinning and lyophilization techniques. The resultant scaffold exhibited favorable biocompatibility and demonstrated the ability to enhance blood clotting, thereby facilitating guided periodontal tissue regeneration. Chitosan-derived biomaterials have been shown to exhibit various beneficial impacts on the regeneration of the heart and blood vessels. In particular, hydrogels containing chitosan demonstrated efficacy in mitigating adverse cardiac remodeling and enhancing cardiac performance in experimental models of cardiomyopathy and myocardial infarction (Domengé et al., 2021; Beleño Acosta et al., 2023). Additionally, specific composite chitosan formulations effectively facilitate electrical conduction, a critical factor in the regeneration of myocardial tissue (Jiang et al., 2019). Chitosan exhibits significant promise in skin regeneration and wound healing due to its antimicrobial and hemostatic characteristics (Ahmed et al., 2018; Abourehab et al., 2022). Gelatin methacryloyl (GelMA) hydrogels containing cell-responsive arginyl-glycyl-aspartic acid (RGD) and matrix metalloproteinases peptide sequences are commonly utilized in tissue engineering due to their flexible mechanical properties, excellent processing capabilities, and exceptional biocompatibility characteristics. Hydrogel microstructures derived from GelMA can be accurately manipulated through contemporary manufacturing methods like 3D printing and electrospinning. Various GelMA hydrogels with diverse microarchitectures have been developed and investigated to replicate the characteristics of the native ECM and regulate the growth, movement, and specialization of different cell populations (Zhang et al., 2022c).

Spinal cord injuries are treated with chitosan-based scaffolds with neural stem cells (NSC). The formation of neurofilament between the scaffold and host tissue was seen by the researcher, which bodes well for further research in the long run (Parvizifard et al., 2020). Thanks to its characteristics, chitosan is frequently employed in products like garakani as an extender or to enhance mechanical and rheological qualities. By combining chitosan, agarose, and cartilage ECM, Garakani created a unique system with sufficient qualities for the tissue engineering of nasal cartilage (Wang et al., 2008).

Zhang et al. (2021) created a thermosensitive hydrogel for skin wound healing by combining oyster peptide microspheres (OPM), β-sodium glycerophosphate (β-GP), and catechol-functionalized chitosan. As per the findings, the hydrogel that has been described quickens the migration of fibroblasts and also speeds up the development of collagen and new blood vessels surrounding the lesion. Additionally, the scientists observed increased total protein (TP) synthesis, suggesting a quicker regeneration process.

An injectable hydrogel with a gelatin foundation that heals itself was created by Vahedi et al. (2020). In particular, combining amylopectin aldehyde groups with gelatin amino groups produced hydrogels that could regain their structure and rheological characteristics. Furthermore, they verified their suitability for scaffolds with osteoinductive qualities in the regeneration of bone tissue (Vahedi et al., 2020).

Tavares et al. (2020) created chitosan/zein composite films with ellagic acid to treat skin infections and speed skin healing. The films that were made had a sufficient thickness that ranged from 133 ± 51 to 283 ± 75 µm. They also demonstrated a sustained drug release of up to 6% after 48 h and a high percentage of water absorption between 114.44 ± 8.07 and 227.94 ± 25.88%. Additionally, films showed antibacterial properties against *P. aeruginosa* and *S. aureus in vitro* (Tavares et al., 2020). To promote wound healing, Maged et al. (2019) created crosslinked-chitosan scaffolds that contained rosuvastatin and were then filled with mesenchymal stem cells (MSCs). Scaffolds demonstrated improved human fibroblast cell proliferation, excellent porosity, and prolonged drug release for 60 hours. An *in vivo* study on Albino rats showed the superiority of MSC-laden scaffolds over plain ones in encouraging wound closure and cell proliferation. Additionally, a histological investigation indicated that scaffolds loaded with stem cells promoted proper collagen distribution in the epidermal layer (Maged et al., 2019).

Miranda et al. (2011) utilized a chitosan-gelatin composite as a framework for cultivating 3D bone marrow mesenchymal stem cells (BMMSCs). The glutaraldehyde crosslinking technique created the porous biocomposite, which enhanced cell adherence, spreading, and vitality. The scaffold exhibited favorable biocompatibility and gradual degradation *in vivo* following its implantation in the tooth sockets of the rat model. The implant remained *in situ* for the full 35 days that the bone healing process took. The chitosan-gelatin composites that are crosslinked exhibit interconnected pores, leading to a reduction in pore size when compared to the gel composites that are not crosslinked. The maximum amount of gelatin needed to achieve 90% cell viability was around 25%; at concentrations more significant than this, such as 50% and 100%, cell viability dropped to less than 40%. Crucially, the crosslinking process boosted the mechanical strength, improved chemical stability, and delayed degradation of the composite scaffolds, all of which improved cell survival (Miranda et al., 2011).

Bio-ink is a substance utilized in 3D printing, comprising living cells and biomaterials designed to replicate the ECM environment. This bio-ink facilitates cell adhesion, proliferation, and differentiation post-printing (Li et al., 2019b; Shen et al., 2020). The extensive usage of chitosan bio-ink in the bioprinting process is demonstrated by the use of the material in the bioprinting of artificial human organs and structures, such as liver or heart valves (Lee et al., 2018; Pisani et al., 2020; Ghahremanzadeh et al., 2021), neural connections (Maturavongsadit et al., 2021), cartilage tissue (Zhao et al., 2020), and bone tissue (Ramirez Caballero et al., 2019).

## 10 Related patents review

Many patents in the field of chitosan and gelatin have been registered in the world. For instance, Patent CN115007114 Chitosan/gelatin composite microsphere is an innovation for water pollution treatment. This innovation highlights the potential of removing heavy metals from water by chitosan/gelatin composite. Patent EP4285737, using chitosan as a food preservative has shown that chitosan inhibits pathogenic and food-spoiling microbes protects food, and increases the life of food products. Another invention, CN116725941, is a chitosan/gelatin/citric acid gel, which belongs to the technical field of drug carriers. This invention uses chitosan and gelatin as cross-linking monomers and citric acid as a cross-linking agent in conditions where the acid content Citric is more than or equal to 0.4% and has good swelling properties, bioavailability, biocompatibility, biodegradability, safety, effective delivery and slow release of hydrophobic drug molecules. This invention realizes a massive potential in the aspect of providing hydrophobic drugs. In patent EP3226923, they made a cartilage gel for cartilage repair, comprising chitosan and chondrocytes. This invention relates to a process for obtaining an implantable cartilage gel for hyaline cartilage tissue repair, consisting of particles from the hydrogel of chitosan and cells capable of forming hyaline cartilage; said process comprises a step of amplification of primary cells in a three-dimensional structure consisting of particles from the physical hydrogel chitosan or chitosan derivative, then a step of redifferentiation and induction of extracellular matrix synthesis by said amplified cells, within the same three-dimensional structure, wherein said cells are primary articular chondrocytes and, or primary mesenchymal stem cells differentiated into chondrocytes. The invention also relates to the resulting cartilage gel and the various uses thereof for cartilage repair following a traumatic impact or an osteoarticular disease such as osteoarthrosis. In another patent, US20240165291, they designed a superabsorbent wound dressing using chitosan. This invention provides a superabsorbent dressing comprising non-woven protonated chitosan fabric/sheet with superior fluid absorption and retention capacity, enhanced tensile strength, and coherency. Patent US20230346726 innovation of soft gelatin capsule that contains ibuprofen drug. This invention relates to a composition for encapsulation in a soft gelatin shell that comprises ibuprofen, one or more polyvinyl pyrrolidone, and one or more polyethylene glycols.

## 11 Conclusion

The knowledge that has lately become accessible about gelatin and chitosan and its uses in tissue engineering, biomedical, food, medicine, water treatment, microencapsulation technology and pharmaceutical management is highlighted in this study. These biomaterials hold great potential for product development owing to their exceptional biodegradability, biocompatibility, antimicrobial, and antioxidant characteristics. Chitosan is a biopolymer that shows promise for food packaging applications due to its capacity to suppress the growth of Gram-negative and Gram-positive bacteria, yeasts, and food-borne filamentous fungi. The favorable attributes of gelatin, such as its ability to biodegrade, biocompatibility, and low toxicity, promote enhanced cell adhesion, differentiation, and proliferation. The distinct qualities of gelatin and chitosan make them popular choices for medication delivery applications. It has been shown that the removal of heavy metals from water by using these bioabsorbents has been effective in water treatment. Consequently, the use of chitosan and gelatin, owing to their distinct attributes, has an extensive range of potential applications across several domains and can significantly contribute to environmental conservation and sustainable growth.

## 12 Future perspectives

Investigating biodegradable polymers, specifically chitosan, and gelatin, shows significant potential for diverse applications in multiple disciplines. Further investigation into chitosan-gelatin composites may result in the creation of sophisticated biomedical products, such as wound dressings, medication delivery systems, and scaffolds for tissue engineering (Huang et al., 2005; Fan et al., 2016; Wang et al., 2016). To satisfy the expanding needs of the healthcare sector, future research may concentrate on improving biocompatibility, optimizing material qualities, and investigating cutting-edge production methods (Bosworth et al., 2019). Using chitosan in packaging applications can potentially reduce environmental pollution on a global scale, notwithstanding various limitations related to thermal stability, barrier properties, mechanical attributes, and manufacturing expenses. Chitosan, when combined with popular cosmetic ingredients like algae extracts, fruit extracts, and essential oils in gel formulations, presents an appealing option for cosmetic products. Chitosan and its derivatives are recommended for incorporation into pharmaceutical formulations intended for slimming products, body weight management, and cosmetics to enhance the efficacy of skin care products and other applications (Morin-Crini et al., 2019). An exciting direction for future study is the creation of biodegradable packaging materials, given the growing emphasis on sustainability and environmental consciousness. Additional research on the characteristics and efficacy of chitosan-gelatin films may open the door to creative packaging options that combine environmental friendliness with practicality (Kan et al., 2019; Wang et al., 2021). Research cooperation amongst scientists from several fields, including biology, materials science, engineering, and food science, will be critical to developing biodegradable polymer-based products. Multidisciplinary research initiatives have the potential to stimulate creativity, promote the sharing of information, and quicken the creation of significant solutions with practical applications (Yin and Yang, 2020). By utilizing their unique qualities and investigating new uses, researchers may help solve urgent social issues, advance sustainability, and enhance human health and wellbeing (Rai et al., 2021; Mukherjee et al., 2023).
